# Exploring the link between SIRT1 gene variants and depression comorbidity in type 2 diabetes

**DOI:** 10.1097/MD.0000000000040563

**Published:** 2024-11-29

**Authors:** Yingxia He, Qinqin Wu, Ziwei Yin, Yi Zeng, Ningyu Xia, Hong Zhu

**Affiliations:** a Department of General Medicine, The Central Hospital of Wuhan, Tongji Medical College, Huazhong University of Science and Technology, Wuhan, Hubei, China.

**Keywords:** correlation analysis, depression, SIRT1 gene, statistics, type 2 diabetes

## Abstract

This study aims to (1) analyze the clinical characteristics and risk factors of patients with type 2 diabetes and comorbid depression and (2) explore the association between SIRT1 gene single-nucleotide polymorphism sites and this comorbidity. A total of 450 type 2 diabetes patients hospitalized in the General Medicine Department at The Central Hospital of Wuhan, Tongji Medical College, Huazhong University of Science and Technology from July 2022 to September 2023, and 300 healthy individuals from the physical examination department were selected as study subjects. Both groups were assessed using general information surveys and questionnaires. Statistical analyses were performed to compare clinical indicators across 3 groups: individuals with only type 2 diabetes, those with comorbid depression, and healthy controls. The age, gender, disease duration, marital status, income and drug expenditure, employment status, fasting blood glucose level, fasting insulin level difference, insulin resistance index difference, glycated hemoglobin, high-density lipoprotein level, and HCY difference among the 3 groups of patients were risk factors for type 2 diabetes comorbid depression patients. The SIRT1 mRNA level was significantly reduced in type 2 diabetes comorbid depression patients. The SIRT1 gene had 3 sites: rs12415800, rs3758391, and rs932658, which were related to the patient’s type 2 diabetes comorbid depression. They were the additive model and dominant model of rs12415800 and rs3758391, respectively. In addition, the GTGGT haplotype composed of rs12415800–rs932658–rs7895833–rs2273773–rs1467568 and the AGACT haplotype composed of rs3758391–rs932658–rs33957861–rs3818292–rs1467568 were significantly associated with type 2 diabetes comorbid depression. Numerous factors influence the presence of depression in patients with type 2 diabetes, with the SIRT1 gene playing a significant role, serving as a potential biomarker for this comorbidity.

## 1. Introduction

Diabetes and depression are 2 major public health problems in today’s society, and their comorbidity has a serious impact on both individual health and social health.^[1]^ There is an interactive relationship between type 2 diabetes and depression. Research shows that patients with diabetes are more likely to suffer from depression, and depression will also aggravate the condition of diabetes. The comorbidity of the 2 causes the prognosis of the disease to worsen, the difficulty and cost of treatment to increase, and the quality of life to decrease.^[2]^ Research showed that the 2 diseases might share common biological and behavioral pathogenesis, such as hypothalamic–pituitary–adrenal axis activation, inflammatory response, insulin resistance, etc.^[3]^ In a genome-wide association study, the rs12415800 polymorphism of the SIRT1 gene was associated with depression, and the rs3758391 locus might be a potential causative factor for depression and plays a regulatory role in controlling SIRT1 gene expression. However, the specific relationship between the SIRT1 gene and type 2 diabetes comorbid depression remains unclear. Existing research showed that there was a two-way link between diabetes and depression, and the comorbidity of the 2 might cause patients to face more challenges and complexities.^[4]^ Global epidemic data showed that the prevalence of diabetes and depression was on the rise, which brought medical and social burdens.^[5]^ Individual factors such as gender, education level, lifestyle, etc are closely related to the risk of type 2 diabetes comorbid depression, suggesting its diverse pathogenesis. Regarding the role of the SIRT1 gene in the occurrence of depression, studies pointed out that it might play an important role in the development of the disease, but its role in type 2 diabetes comorbid depression required in-depth study. Therefore, this study used relevant experiments to explore the correlation between SIRT1 gene polymorphisms and disease status to provide a more accurate reference for clinical diagnosis and treatment.

## 2. Materials and methods

### 2.1. General information

#### 2.1.1. Research objects

This study was approved by The Ethics Committee of The Central Hospital of Wuhan. Four hundred fifty patients with type 2 diabetes who visited The Central Hospital of Wuhan, Tongji Medical College, Huazhong University of Science and Technology Department of general medicine from July 2022 to September 2023 were selected, and 300 healthy people (diabetes and depression were excluded) who had physical examinations in the physical examination department were selected as controls. Research questionnaires on experimental subjects and corresponding disease treatment data were collected. The experimental subjects were divided into healthy control group, type 2 diabetes without depression group, and type 2 diabetes comorbid depression group. Inclusion criteria: experimental subjects in all groups are aged between 18 and 65 years old, and have signed an experimental informed agreement, and are individuals who voluntarily participate in this experimental study. Healthy control group: do not meet the diagnostic criteria for type 2 diabetes and have a PHQ-9 score < 5 points. Diabetes without depression group: meet the diagnostic criteria for type 2 diabetes proposed by the World Health Organization Expert Committee on Diabetes (1999) and have a PHQ-9 score of < 5 points. Type 2 diabetes comorbid depression group: meet the diagnostic criteria for type 2 diabetes and have a PHQ-9 score of ≥ 5 points. Exclusion criteria: (1) patients have a history of mental illness, have received psychological treatment, or have experienced other major illnesses. (2) Patients who cannot accurately describe their feelings and cannot cooperate in completing the experiment. (3) Pregnant and lactating women.

The entire experiment conducted in the study has been approved by the ethics committee of our hospital, and all patients have signed informed consent forms.

#### 2.1.2. Data collection

General information questionnaire: record the age, gender, marital status, education, employment status, income, number of prescription drugs, drug expenditures, duration of diabetes, hypoglycemic plan, diabetes complications, family history of diabetes, tobacco and alcohol history, etc of all participants.

Collection of clinical indicators: height, weight, waist circumference, and blood pressure. Collect fasting blood, conduct blood lipid tests (triglycerides, total cholesterol, high-density lipoprotein cholesterol, low-density lipoprotein cholesterol), and measure fasting blood sugar, fasting insulin, glycated hemoglobin, and HCY.

### 2.2. Research methods and steps

#### 2.2.1. Depression score measurement

The PHQ-9 depression scale was used to measure the depression scores of experimental subjects. Patients with a PHQ-9 score of ≥ 5 points were considered to be depressed, and patients with a PHQ-9 score of < 5 points were considered to have no depression. The test results of the patients’ PHQ-9 depression scale were used as the evaluation standard for whether the patients suffered from depression, and the patients were divided into groups based on this. The Hamilton Depression Rating Scale (HAMD) and the Hamilton Anxiety Rating Scale (HAMA) were used to evaluate the depression and anxiety of experimental subjects in each group to ensure the accuracy of the experimental results.

#### 2.2.2. SIRT1mRNA research

(1) Sample collection: collect approximately 5 mL of fasting elbow vein blood from the patient using anticoagulant blood collection vessels. (2) Serum preparation: within 2 hours, centrifuge the blood sample to obtain serum, and then store the serum in an environment of −80 °C. (3) Sample processing: wash the serum with precooled PBS buffer, add 1 mL of TRIzol reagent, and transfer it to a centrifuge tube without mRNA enzyme. (5) Detection of RNA concentration: use a spectrophotometer to measure the mRNA concentration of the sample. (6) Reverse transcription: add 10 µL buffer, 2 µL mRNA template, 1.2 µL primers, 0.2 µL reverse transcriptase, and mRNA free water to 20 µL in a new centrifuge tube for reverse transcription. (7) PCR analysis: add 10 µL of PCR reagent, 3 µL of upstream and downstream primers, 1 µL of cDNA, and 3 µL of distilled water to a small centrifuge tube, and then use a PCR instrument for analysis. (8) Data analysis: after the experiment, use Excel software to calculate the relative expression of mRNA. Analyze the relationship between the clinical characteristics of patients and the difference of mRNA SIRT1 level to evaluate the possibility of SIRT1 level as a diagnostic marker of depression in diabetes patients.^[6]^

#### 2.2.3. SIRT1 gene polymorphisms

Download the sequence and frequency of the SIRT1 gene from the 1000Genomes website, and then use the paired labeling method with Haploview 4.1 software to select the single-nucleotide polymorphism (SNP) sites of the SIRT1 gene. When selecting, set *R* ≥ 0.8 and minimum allele frequency ≥ 0.1. Use the Hardy–Weinberg law to evaluate the frequency and inheritance pattern of SIRT1 gene.

The first-generation (Sanger) sequencing method was used and the kit produced by Sangon Biotechnology Company was used to extract DNA from the patient’s peripheral blood. The amplified PCR product was detected through PCR amplification, agarose electrophoresis detection, gel recovery, and other steps. The PCR products were purified and sequenced using the 3730XL sequencer produced by the American ABI Company. The specific steps are as follows: collect 1 mL of fasting venous blood from the subjects, use the blood gene kit developed by Sangon Biotech to extract the DNA of the blood genome, and screen out 11 SNP in the SIRT1 gene loci for genotyping. Primer Premier 5 software was used to design primers for each site in the SIRT1 gene, and the primers were provided by Sangon Biotech. The PCR reaction system includes 1 µL template DNA, 2 µL upstream and downstream primers F and D, 1 µL Dntp, 2.5 µL Taq Buffer (with MgCl_2_), 0.2 Taq enzyme, and 25 µL deionized water. The setting conditions of the reaction are as follows: first, pre-denature (denaturation step) for 5 minutes at 95 °C, followed by 10 cycles, with each cycle holding at 94 °C for 30 seconds (denaturation) and 63 °C for 30 seconds (annealing) and holding at 72 °C for 30 seconds (extension). This was followed by 30 cycles, each consisting of 30 seconds at 95 °C (denaturation), 30 seconds at 58 °C (annealing), and 30 seconds at 72 °C (extension). Finally, after repair and extension at 72 °C for 10 minutes, heat preservation was performed at 4 °C. Five microliters of 1% agarose gel electrophoresis was taken to detect the obtained PCR products. The corresponding bands that met the requirements were cut, recovered, and sent to Sangon Biotechnology Company for sequencing. Sequencing results were analyzed using sequence analysis software, and sequence alignment was analyzed using SeqMan software.

#### 2.2.4. Linkage disequilibrium analysis

Haploview software was used to perform linkage disequilibrium analysis on the detected SNP sites. The typing results showed the polymorphism distribution of the SIRT1 gene in the population and its correlation with type 2 diabetes comorbid depression.

#### 2.2.5. Haplotype analysis

Blocks with strong correlations were found based on the results of linkage disequilibrium analysis, and then haplotypes were constructed for analysis.

#### 2.2.6. Statistical processing

SPSS26.0 statistical software was used for data analysis. The measurement data that conformed to the normal distribution were expressed as (x ± s), and the grouped *t* test was used for comparison between the 2 groups. The measurement data that did not conform to the normal distribution were expressed as M (P25, P75), and the nonparametric test was used for the comparison between the groups. Count data were expressed as relative numbers, and tests were used for comparisons between groups *x*^2^. First, single-factor logistic regression analysis was used to screen out possible relevant variables, and then multi-factor logistic regression analysis was used to determine the influencing factors of type 2 diabetes comorbid depression. The significance level was set to a *P*-value <.05, that is, *P* < .05, indicating that the difference was statistically significant.

#### 2.2.7. Quality control

The investigation plan was jointly designed by experts from the general medicine department, endocrinology department, and neurology department of Wuhan Central Hospital. Professional learning and training were conducted before officially launching the investigation. Patients and investigators were required to fill in the questionnaire honestly and objectively, and the questionnaires were randomly checked regularly to evaluate the authenticity and reliability of the questionnaires. The person in charge provided support at all times to ensure the quality of the investigation. Finally, the survey data were compiled and the entered data were verified by a dedicated person.

## 3. Results

### 3.1. Patient clinical record information

According to the PHQ-9 depression scale, among the 450 patients with type 2 diabetes, 80 patients with type 2 diabetes without depression, 70 patients with type 2 diabetes comorbid depression, and 300 healthy peoples served as controls. Patients with type 2 diabetes without depression were coded as T2DM, patients with type 2 diabetes comorbid depression were coded as T2DD, and healthy peoples were coded as HP. A comparison of the baseline data of the 3 groups of experimental subjects is shown in Table 1.

**Table 1:** Comparison of experimental subjects baseline data [N = 250, x ± s, M (P25, P75)].

Project	T2DM (240 cases)	T2DD (210 cases)	HP (300 cases)	*P*-values
Age (years)*	45.00 (41.24, 46.32)	52.00 (49.36, 54.85)	38.00 (36.21, 42.21)	<.001
Gender (male/female)†	135/105	150/60	156/144	<.001
Marital status (married/unmarried)†	238/12	195/15	164/36	<.001
Education level (illiterate/below undergraduate/above undergraduate)†	30/150/60	66/66/78	24/219/97	.072
Employment situation (unemployed/employed/retired/retired)†	9/231/0/0	21/186/0/3	30/270/0/0	.004
Income (yuan/month)*	4100 ± 1000	3000 ± 200	4000 ± 1000	<.001
Prescription medication quantity (1/2/3/4)†	75/105/33/27	42/84/69/15	–	.065
Monthly medication expenses (yuan/month)*	200±100	500±100	50±100	<.001
Disease course (year)‡	2.00 (1.65,2.31)	4.00 (3.65,4.21)	–	<.001
Family history of diabetes (yes/no)†	63/177	39/171	108/192	.081
History of smoking (with/without)	117/123	87/123	168/132	.054
History of drinking (with/without)	105/135	96/114	153/147	.215

* Single-factor analysis x ± s.

† Chi-square test.

‡ M (P25, P75) test.

From Table 1, there was no statistically significant difference in the education level, number of prescription drugs, family history of diabetes, smoking history, and drinking history among the 3 groups of experimental subjects (*P* > .05). The difference of age, gender, marital status, disease duration, income, drug expenditure, and employment status of the 3 groups of patients were all statistically significant (*P* < .05). From the perspective of age, T2DD > T2DM > HP, indicating that the older patients are, the more likely they were to suffer from type 2 diabetes comorbid depression. From the perspective of the male-to-female ratio, T2DD > T2DM > HP, indicating that men were more likely to suffer from type 2 diabetes comorbid depression. From the perspective of marital status, T2DD > T2DM ≈ HP, indicating that marriage had no impact on diabetes, but married patient were more likely to suffer from depression. From the perspective of disease course, T2DD > T2DM, indicating that type 2 diabetes had a longer duration of comorbid depression. The clinical characteristics of the 3 groups of patients are shown in Table 2.

**Table 2:** Comparison of clinical characteristics of 3 groups of experimental subjects [N = 250, x ± s, M (P25, P75)].

Project	T2DM (240 cases)	T2DD (210 cases)	HP (300 cases)	*P*-values
Height (m)	1.62 ± 0.04	1.60 ± 0.03	1.61 ± 0.05	>.05
Weight (kg)	62.34 ± 10.35	61.34 ± 11.54	62.99 ± 10.98	.132
Body mass index (kg/m^3^)	24.31 ± 2.14	22.11 ± 1.98	23.11 ± 2.00	.073
Waist (cm)	85.24 ± 5.36	86.11 ± 4.96	85.58 ± 5.01	.182
Systolic pressure (mm Hg)	117.65 ± 14.65	118.65 ± 14.62	116.32 ± 12.32	.186
Diastolic pressure (mm Hg)	82.34 ± 14.65	81.24 ± 15.64	82.45 ± 14.11	.117
Fasting blood glucose levels (mmol/L)	6.11 (6.01, 6.24)	7.65.01 (7.41, 7.85)	5.01 (4.36, 5.21)	.001
Differences in fasting insulin levels (mU/L)	11.63 (10.25, 12.01)	9.12 (8.63, 11.20)	6.69 (6.54, 6.75)	.092
Glycated hemoglobin level (mmol/L)	8.10 (7.63, 8.32)	7.14 (6.32, 7.65)	5.01 (4.21, 5.43)	<.001
Cholesterol levels (mmol/L)	4.62 (4.01, 4.86)	4.52 (4.21, 4.75)	4.54 (4.14, 4.65)	.171
Triglyceride levels (mmol/L)	1.75 (1.63, 1.84)	1.66 (1.54, 1.75)	1.71 (1.66, 1.79)	.084
High-density lipoprotein levels (mmol/L)	0.98 (0.65, 1.11)	1.11 (1.00, 1.21)	1.33 (1.11, 1.54)	<.001
Low-density lipoprotein levels (mmol/L)	3.10 (2.86, 3.24)	3.01 (2.88, 3.14)	3.05 (2.90, 3.12)	.375
HCY (µmol/L)	15.36 ± 5.63	1 4.99 ± 5.12	7.63 ± 3.63	<.001

From Table 2, there was no statistically significant difference in height, weight, waist circumference, blood pressure (systolic blood pressure and diastolic blood pressure), cholesterol, triglyceride, and low-density lipoprotein levels among the 3 groups of experimental subjects. The difference of fasting blood glucose, fasting insulin, glycated hemoglobin, high-density lipoprotein, and HCY were statistically significant. From the perspective of fasting blood glucose levels, T2DD > T2DM > HP. From the perspective of fasting insulin difference, T2DM > T2DD > HP. From the perspective of glycated hemoglobin level, T2DM > T2DD > HP. Judging from high-density lipoprotein levels, T2DM < T2DD < HP. From the perspective of HCY content, T2DM ≈ T2DD > HP.

### 3.2. Comparison of experimental subjects’ HAMD and HAMA scores

**Figure 1.**
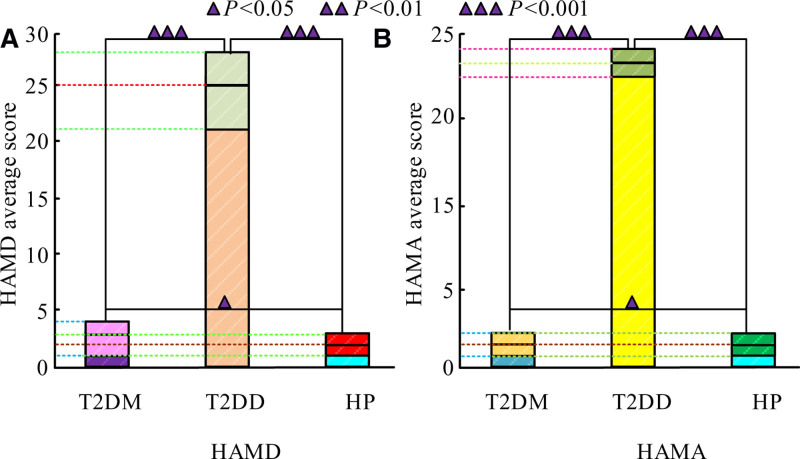
Score results of HAMD and HAMA in 3 groups of experimental subjects. HAMA = Hamilton Anxiety Rating Scale, HAMD = Hamilton Depression Rating Scale.

The 3 groups of experimental subjects were evaluated using HAMD and HAMA, respectively. The results are shown in Figure 1.

There were differences in the HAMD and HAMA scores among the 3 groups of experimental subjects. As shown in Figure 1A, the HAMD score of T2DM was 3.00 (1.00, 4.00), the HAMD score of T2DD was 25.00 (21.00, 28.00), and the HP score was 2.00 (1.00, 3.00). It showed that the degree of depression of patients in the T2DD group was much greater than that of the other 2 groups, which verified the classification results of the PHQ-9 depression scale. In Figure 1B, the HAMD score of T2DM was 1.50 (0.70, 2.80), the HAMD score of T2DD was 23.00 (22.00, 24.00), and the HP score was 1.50 (0.70, 2.80). This showed that the anxiety level of patients in the T2DD group was much greater than that of the other 2 groups. Next, logistic regression univariate analysis was performed on the experimental subjects’s age, gender, disease course, marital status, income and medication expenses, employment status, fasting blood glucose level, fasting insulin level, insulin resistance index, glycated hemoglobin, high-density lipoprotein level, and HCY level. The results are shown in Table 3.

**Table 3:** Logistic regression univariate analysis of T2DD risk factors.

	Variable	B	SE	Wald	df	*P*	OR	95% confidence interval	
								Lower limit	Upper limit
Gender	Female	–	–	–	–	–	1	–	–
	Male	0.91	0.16	33.68	1	<.05	2.47	1.82	3.36
	Constant	–	–	–	–	<.05	0.24	1.01	1.04
Age	<40	–	–	–	–	–	11.41	–	–
	>40	1.41	0.49	8.45	1	<.05	4.10	1.58	10.61
	Constant	-2.15	0.47	20.74	1	<.05	0.12	–	–
Course of disease	–	0.03	0.03	8.35	1	<.05	1.03	1.01	1.05
	Constant	-1.27	0.12	107.10	1	<.05	0.28	–	–
Marital status	Unmarried	–	–	–	1	–	–	–	–
	Married	0.43	0.50	0.72		<.05	1.53	0.57	4.10
	Constant	-1.48	0.49	8.94	1	<.05	0.22		
Medical expenses	Income	1.95	0.75	6.68	1	<.05	7.04	1.60	30.93
	Expenditure	1.34	0.62	4.61	1	<.05	3.81	1.12	12.95
	Constant	-1.47	0.18	68.76	1	<.05	0.23		
Employment situation	Unemployed	-0.03	0.16	0.06	1	<.05	0.98	0.71	1.3
	Employed	–	–	–	–	–	–	–	–
	Retired	–	–	–	–	–	–	–	–
	Constant	-0.90	0.13	54.88	1	<.05	0.37	–	–
Clinical indicators	Fasting blood glucose levels	0.58	0.20	8.72	1	<.05	1.80	1.21	2.66
	Constant	1.10	0.08	9.33	1	<.05	0.33	–	–
	Fasting insulin level	0.42	0.56	0.57	1	<.05	1.53	0.50	4.61
	Constant	1.10	0.08	69.33	1	<.05	–	–	–
	HOMA IR	0.01	0.03	5.11	1	<.05	1.07	1.01	1.14
	Constant	-1.66	0.30	30.17	1	<.05	0.19	–	–
	Glycosylated hemoglobin	0.39	0.39	5.90	1	<.05	1.47	1.07	2.02
	Constant	-1.25	0.13	92.77	1	<.05	0.286	–	–
	High-density lipoprotein levels	0.11	0.07	4.077	1	<.05	1.12	1.00	1.25
	Constant	-1.16	0.12	92.29	1	<.05	0.31	–	–
	HCY	-0.036	0.022	2.707	1	<.05	0.964	0.923	1.007
	Constant	-0.065	0.575	0.013	1	<.05	0.937	–	–

According to the results in Table 3, the experimental subjects’s age, gender, course of disease, marital status, income and medication expenses, employment status, fasting blood glucose level, fasting insulin level, insulin resistance index, glycated hemoglobin, high-density lipoprotein level, and HCY level are risk factors for T2DD. Taking gender as an example, male patients were associated with type 2 diabetes patients with depression (OR = 2.473, CT = 1.82–3.36, <0.05).

### 3.3. Expression and identification of SIRT1 mRNA in different experimental subjects

**Figure 2.**
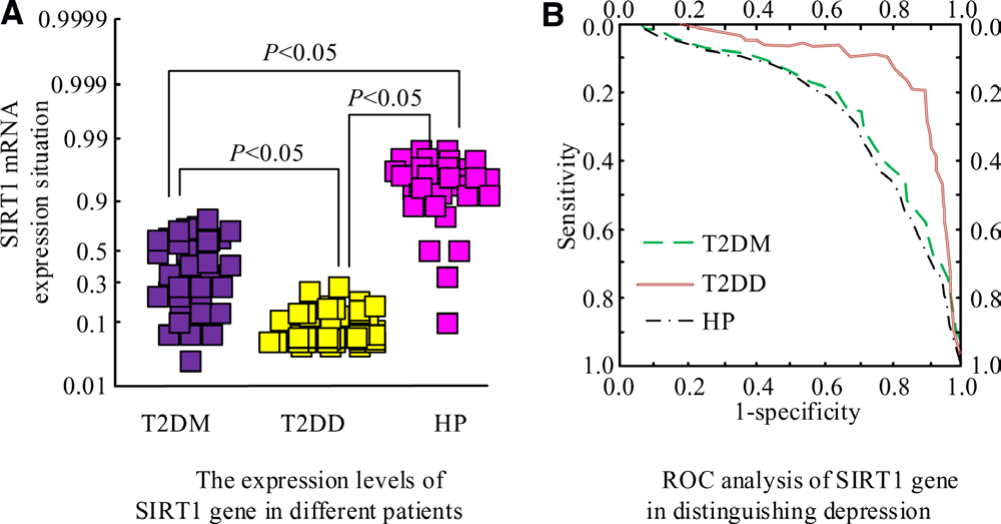
Expression and identification results of SIRT1 gene in 3 groups of experimental subjects.

In Figure 2A, there was a statistically significant difference in the expression of SIRT1 mRNA among the 3 groups. Subsequently, pairwise comparisons between groups revealed that compared with the HP group, the SIRT1 mRNA expression in both T2DM and T2DD groups decreased, with statistically significant differences. Compared with the T2DM group, the expression of SIRT1 mRNA in the T2DD group decreased, and the difference was statistically significant. There was a strong association between mRNA expression and type 2 diabetes comorbid depression. The ROC analysis of using SIRT1 gene to identify experimental subject cases is shown in Figure 2B. The AUC value of T2DD was 0.914, the AUC value of HP was 0.721, and the AUC value of T2DM was 0.701, indicating that the extraction of SIRT1 gene could be used to identify healthy people and type 2 diabetes. No depression, type 2 diabetes comorbid depression patients had better abilities.

### 3.4. Correlation between SIRT1 gene expression and experimental subjects HAMD and HAMA scores

**Figure 3.**
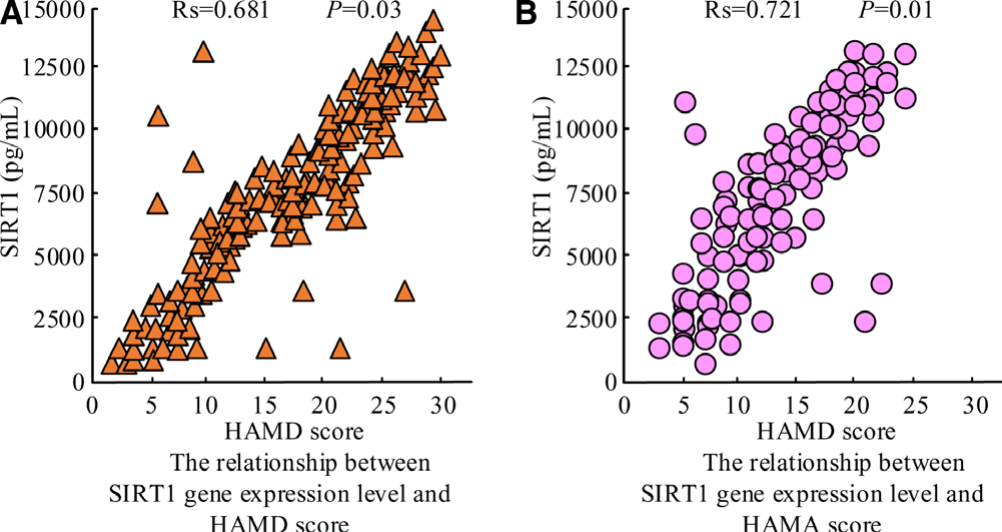
Relationship between SIRT1 gene expression and HAMD and HAMA scores. HAMA = Hamilton Anxiety Rating Scale, HAMD = Hamilton Depression Rating Scale.

The correlation analysis between the experimental subject’s SIRT1 gene expression and HAMD and HAMA scores was performed. The results are shown in Figure 3. From Figure 3A, the patient’s SIRT1 gene expression was positively correlated with the HAMD score, and the correlation coefficient was 0.681, which was statistically significant. From Figure 3B, the patient’s SIRT1 gene expression was positively correlated with the HAMA score, and the correlation coefficient was 0.721, which was statistically significant.

### 3.5. Allele and genotype frequency analysis of SIRT1 gene SNP sites

The study detected a total of 11 SNP sites of the SIRT1 gene in patients with type 2 diabetes without depression and patients with type 2 diabetes comorbid depression. Chi-square tests were performed on the genotype models and corresponding allele frequencies of these sites. Among the SNP sites of these 11 SIRT1 genes, the SNP sites with significant differences in genotype frequencies in the co-dominant model were rs12415800, rs3758391, and rs932658. The SNP sites with significant differences in the dominant model were rs12415800 and rs3758391. The results are shown in Table 4

**Table 4:** Distribution frequencies of alleles and genotypes in SNP sites of SIRT1 gene.

SNP	Model	Genotype	T2DM	T2DD	*x* ^2^	OR	*P*
rs12415800	Co display model	GA	4	2	8.213	–	.018
		GG	19	76			
		AA	199	432			
	Explicit model	GA + GG	16	45	1.362	0.863 (0.452–1.123)	.179
		AA	163	489			
	Recessive model	GG	4	1	2.534	2.463 (1.635–4.652)	.067
		GA + AA	210	720			
	Allele	G	21	84	0.756	0.812 (0.512–1.258)	.463
		A	410	963			
rs3758391	Co display model	GA	3	7	7.653	–	.043
		GG	24	105			
		AA	186	532			
	Explicit model	GA + GG	24	110	4.623	0.653 (0.389–0.963)	.003
		AA	186	410			
	Recessive model	GG	4	3	0.965	2.072 (0.462–9.653)	.354
		GA + AA	245	605			
	Allele	G	30	114	2.634	0.670 (0.486–0.964)	.154
		A	412	902			
rs932658	Co display model	CC	2	22	6.321	–	.365
		TC	76	165			
		TT	70	210			
	Explicit model	CC + TC	74	205	0.013	0.998 (0.763–1.365)	.002
		TT	175	563			
	Recessive model	CC	1	21	6.120	2.312 (0.465–9.365)	.364
		TT + TG	210	623			
	Allele	C	71	224	0.563	0.894 (0.761–1.032)	.142
		T	367	994			

SNP = single-nucleotide polymorphism.

In Table 4, the rs12415800 locus was associated with type 2 diabetes comorbid depression under the co-dominant inheritance model, and the GG genotype might be a risk factor for type 2 diabetes comorbid depression under the recessive inheritance model. The risk of patients carrying the GG genotype to develop depression (compared to patients with the GA and AA genotypes) was 2.463 times, with a 95% confidence interval of (1.635–4.652). It showed that the rs12415800 locus was associated with the occurrence of type 2 diabetes comorbid depression, especially in patients with the GG genotype, who had a higher risk of depression. Similarly, the rs3758391 locus was associated with type 2 diabetes comorbid depression under the co-dominant inheritance model, which also showed that patients with the GG genotype had a higher risk of depression. The rs932658 locus was associated with type 2 diabetes comorbid depression in the dominant model. Under the dominant model, CC + TC was a protective factor for type 2 diabetes comorbid depression, and patients with type 2 diabetes with TT genotype had a higher risk of depression.

### 3.6. Association analysis of SIRT1 gene SNP sites and type 2 diabetes comorbid depression

The study used logistic regression analysis to analyze 11 SNP sites in the SIRT1 gene and type 2 diabetes comorbid depression. It showed that 3 sites, rs12415800, rs3758391, and rs932658, were related to the onset of depression in patients, as shown in Table 5.

**Table 5:** Logistic regression analysis of 3 SNP sites of SIRT1 gene.

SNP	Model	Genotype	T2DM	T2DD	OR	*P*
rs12415800	Additive model	GA	4	2	0.963 (0.642–1.365)	.463
		GG	19	76		
		AA	199	432		
	Explicit model	GA + GG	16	45	0.863 (0.452–1.123)	.180
		AA	163	489		
	Recessive model	GG	4	1	8.369 (5.635–9.652)	.056
		GA + AA	210	720		
	Allele	G	21	84	0.812 (0.512–1.258)	.421
		A	410	963		
rs3758391	Additive model	GA	3	7	0.612 (0.456–0.712)	.456
		GG	24	105		
		AA	186	532		
	Explicit model	GA + GG	24	110	0.653 (0.389–0.963)	.004
		AA	186	410		
	Recessive model	GG	4	3	6.072 (0.462–9.653)	.254
		GA + AA	245	605		
	Allele	G	30	114	0.670 (0.486–0.964)	.153
		A	412	902		
rs932658	Additive model	CC	2	22	1.023 (0.965–1.124)	.034
		TC	76	165		
		TT	70	210		
	Explicit model	CC + TC	74	205	0.998 (0.763–1.365)	.004
		TT	175	563		
	Recessive model	CC	1	21	6.312 (5.465–10.687)	.364
		TT + TG	210	623		
	Allele	C	71	224	0.894 (0.761–1.032)	.142
		T	367	994		

SNP = single-nucleotide polymorphism.

After adjusting for the patient’s age, gender, and other factors, for the rs932658 locus, under the recessive model, the risk of depression in type 2 diabetes patients with CC and TC genotypes was reduced by 46.3% compared with patients with TT genotypes. It indicated that rs932658 was a protective factor for type 2 diabetes comorbid depression. For the rs12415800 site and rs3758391 site, under the additive model and the dominant model, patients with type 2 diabetes with GG genotype had a higher risk of depression than patients with GA and AA genotypes. Therefore, the rs12415800 site and rs3758391 site were closely related to type 2 diabetes. There was a significant association with comorbid depression in patients with diabetes.

### 3.7. Linkage disequilibrium analysis of SNP sites of SIRT1 gene

**Figure 4.**
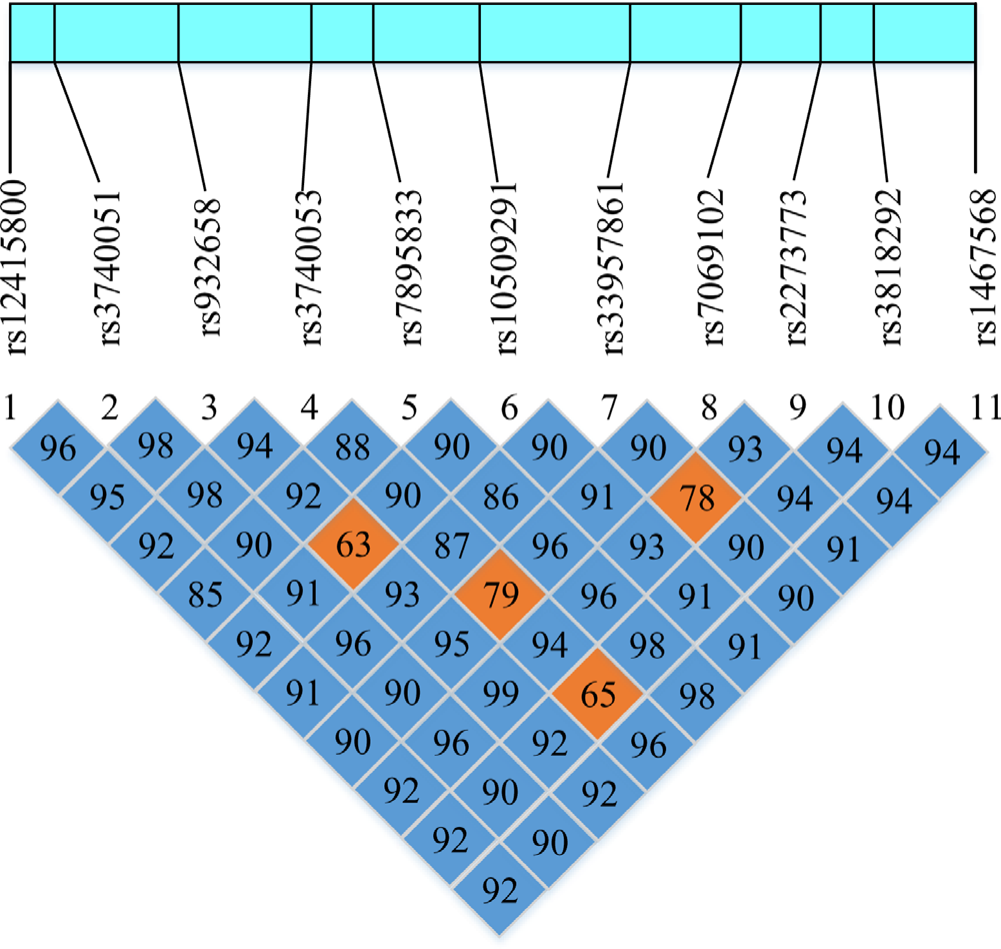
Linkage disequilibrium analysis diagram of 11 SNP sites of SIRT1 gene. SNP = single-nucleotide polymorphism.

The linkage disequilibrium diagram studying the typing results of 11 SNP sites in the SIRT1 gene is shown in Figure 4. Comparing the typing results with the SIRT1 gene linkage disequilibrium analysis results in the database, the data matching between the 2 was relatively high, indicating that the typing results had extremely high credibility.

### 3.8. Haplotype association analysis of SIRT1 gene SNP sites

The results of SIRT1 gene haplotype association analysis are shown in Table 6.

**Table 6:** SIRT1 gene haplotype association analysis results.

SNP site	Haplotype	Type 2 diabetes without depression	Type 2 diabetes comorbid depression	*P*	OR (95% CI)
rs12415800–rs932658–rs7895833-rs2273773–rs1467568	AAC	0.356	0.328	.365	1.133 (0.883–1.452)
	GGA	0.192	0.189	.864	1.020 (0.714–1.458)
	CAC	0.617	0.168	1.658	1.132 (0.780–1.642)
	CGG	0.630	0.587	2.698	0.775 (0.527–1.140)
	GGTGA	0.648	0.655	.698	1.139 (0.785–1.651)
	GTGGT	0.668	0.652	.001	1.168 (0.835–1.633)
	GTGCC	0.083	0.108	.190	1.062 (0.830–1.357)
rs3758391–rs932658–rs33957861–rs3818292–rs1467568	GGAA	0.272	0.264	.359	1.197 (0.800–1.790)
	AGACT	0.341	0.345	.002	1.039 (0.809–1.333)
	GTGGT	0.312	0.176	.494	0.985 (0.779–1.244)
	GTGCC	0.098	0.606	.267	1.153 (0.904–1.472)
	GGGTGC	0.034	0.161	.173	1.815 (0.766–4.297)
rs932658–rs3740053–rs7895833–rs33957861–rs2273773	GGG	0.606	0.550	.494	1.118 (0.776–1.612)
	GGA	0.192	0.155	.195	0.297 (0.144–0.610)
	GCTAA	0.144	0.196	.324	0.775 (0.527–1.140)
	GTGCT	0.648	0.142	.914	1.197 (0.800–1.790)
	AGACT	0.116	0.267	.155	1.039 (0.809–1.333)

SNP = single-nucleotide polymorphism.

From the haplotype analysis in Table 5, 2 of the haplotypes composed of SIRT1 gene SNP sites were related to depression in patients. They were GTGGT composed of rs12415800–rs932658–rs7895833–rs2273773–rs1467568 (*P* = .001) and AGACT composed of rs3758391–rs932658–rs33957861–rs3818292–rs1467568 (*P* = .002). Patients with type 2 diabetes who carried GTTGGT and AGACT haplotypes had a higher risk of comorbid depression than patients with other haplotypes, indicating that these 2 haplotypes were potential pathogenic factors for type 2 diabetes comorbid depression.

## 4. Discussion

### 4.1. Analysis of clinical characteristics of type 2 diabetes comorbid depression

From early medical observations to recent research results, the proportion of patients with diabetes comorbid with depression was significantly higher than that of patient without diabetes.^[7]^ The onset of type 2 diabetes comorbid depression is affected by many factors. Research results showed that older patients had a greater proportion of diabetes and depression. Male patients were more likely to suffer from depression than female patients. Married patient were more likely to suffer from depression than unmarried patient. Because older patients’ physical functions gradually weaken, their ability to cope with the disease also decreases, increasing the risk of diabetes and depression. When faced with stress, men are often more reluctant to express emotions and seek help, increasing their risk of depression. Married patient face more social pressure, work pressure, etc, increasing the risk of depression. Therefore, in preventing and treating diabetes and depression, individual physiological, psychological and social factors should be comprehensively considered, and effective measures should be taken to reduce the risk of disease.^[8]^

### 4.2. Significant reduction of SIRT1 mRNA expression in type 2 diabetes comorbid depression patients

Data showed that the expression of certain sites of the SIRT1 gene was related to the onset of depression. A case study showed that 91 patients with depression and 85 healthy people were compared with SIRT1 mRNA levels in the blood to evaluate the differences between the 2 groups.

As a result, patients with depression had lower levels of SIRT1 mRNA in the blood.^[9]^ In another case data, researchers analyzed the anthropometric and biochemical indicators of 127 adults and used TaqMan kits to determine the genotypes of rs3758391 and rs1800470. The expression of SIRT1 and TGF-β1 was analyzed by real-time PCR. The results showed that compared with patients carrying the CC genotype, the gene expression of SIRT1 and TGF-β1 increased by 1.8 ± 0.6-fold and 1.3 ± 0.6-fold, respectively, in patients carrying the TT genotype of rs3758391 and rs1800470.^[10]^ This study used PCR to detect the expression of SIRT1 mRNA in peripheral blood and found differences among the 3 groups. Compared with the healthy group, the expression of SIRT1 mRNA in type 2 diabetes without depression group and type 2 diabetes comorbid depression group decreased, which was consistent with the expected results, indicating that SIRT1 played a role in type 2 diabetes comorbid depression. Compared with type 2 diabetes without depression group, the expression of SIRT1 mRNA in type 2 diabetes comorbid depression group was lower, indicating that the expression of SIRT1 mRNA was related to type 2 diabetes comorbid depression. The study speculated that the possible mechanism was that the decrease of SIRT1 expression led to the aggravation of insulin resistance and inflammatory reaction, which led to the occurrence of type 2 diabetes comorbid depression. In type 2 diabetes patients, due to the down-regulation of SIRT1 expression level, the neuroprotective effect was reduced, and depressive behaviors were increased, which further led to type 2 diabetes comorbid depression. Since current research on SIRT1 gene expression in patients with type 2 diabetes is limited, further research is needed. In addition, the ability of the SIRT1 gene as a disease marker was gradually developed. A cross-sectional study involving 120 diabetic patients showed significant changes in the expression of the SIRT1 gene in peripheral blood and was correlated with the degree of depression (*R*s = 0.452; *P* = .003), which indicated that the SIRT1 gene became a potential biomarker for assessing the onset of depression in patients with diabetes.^[11,12]^ The experimental results of the research showed that using the SIRT1 gene had higher accuracy in identifying patients with type 2 diabetes comorbid depression, and the AUC value of the ROC analysis chart was 0.914. However, its specific application in disease diagnosis and treatment still requires more in-depth research to solve this problem. It is hoped that through further research, the SIRT1 gene can be used as a differential marker for depression in patients with diabetes, providing a more effective method for clinical diagnosis and treatment, thereby achieving early diagnosis and treatment of the disease.

### 4.3. The different distributions of SIRT1 gene SNP sites

The study selected 11 SNP sites of the SIRT1 gene and conducted correlation analysis to explore whether there were differences in the distribution frequencies of SNP site genotypes and alleles in patients with type 2 diabetes and type 2 diabetes comorbid depression. Therefore, the association between the SNP sites of the SIRT1 gene and depression in patients was explored. The research results showed that the SNP sites with significant differences in genotype frequencies in the co-dominant model were rs12415800, rs3758391, and rs932658. The SNP sites with significant differences in the dominant model were rs12415800 and rs3758391. The distribution frequencies of genotypes and alleles in the 3 models were significantly different, which further illustrated that the distribution frequencies of genotypes and alleles of the SNP sites of the SIRT1 gene in patients with type 2 diabetes and type 2 diabetes comorbid depression were different.

### 4.4. Correlation analysis

Logistic regression analysis found that these 3 loci, rs12415800, rs3758391, and rs932658, were related to the onset of depression in patients. Among them, 2 loci, rs12415800 and rs3758391, were shown to be related to the degree of depression in patients in the additive model and the dominant model. The study speculated that rs12415800 and rs3758391 were the potential causative factors of depression. At the same time, there was a significant association between single-nucleotide polymorphisms of the SIRT1 gene and major depressive disorder. Ninety-two healthy participants were selected from eastern China. Through image segmentation, the gray matter density of each voxel was obtained, and then a multiple linear regression framework was used to determine the impact of SIRT1 SNPs on gray matter density. It was speculated that the SIRT1 gene might be a factor leading to susceptibility to major depressive disorder.^[13]^ Other data showed that the protein encoded by the SIRT1 gene affected DNA damage repair, cell apoptosis, and circadian rhythm through multiple pathways, thereby affecting the function of the central nervous system and leading to the occurrence of depression.^[10,14]^ Therefore, in patients with type 2 diabetes, especially those with comorbid depression, changes in SIRT1 might play a regulatory role in the brain of patients with depression, affecting emotion regulation and cognition by affecting the function of the central nervous system.^[15]^

### 4.5. SIRT1 is based on the regulatory mechanism of type 2 diabetes with depression

The SIRT1 gene is a gene encoding the SIRT1 protein, which is an NAD + dependent deacetylase that plays various roles in regulating metabolism, stress response, and cell cycle in cells. Because type 2 diabetes patients often have metabolic disorders. The SIRT1 gene can affect the occurrence of depression by regulating the activity of glucose metabolism and lipid metabolism pathways. SIRT1 also plays an important protective role in the nervous system, including neuronal protection and regulation of synaptic plasticity. Depression is closely related to neurological dysfunction, and the neuroprotective effect of SIRT1 may help alleviate the occurrence and development of depression.

Depression has a negative impact on patients’ health and quality of life. Particularly in patient with type 2 diabetes, depression can worsen the condition when other risk factors are present. By detecting polymorphic changes in specific gene loci, patient at risk of depression can be screened out. Early psychological intervention and necessary drug treatment can be provided to reduce the incidence of depression and even to prevent the occurrence of depression. However, the experiment has some limitations. First, the experimental subjects selected are only patients in 1 hospital. Patients in 1 hospital cannot represent all patients with type 2 diabetes, which limits the breadth and practicality of the results. In addition, as an observational study, the experimental results are inevitably subject to chance and bias. Therefore, future research needs to select large-scale samples for experiments and increase the diversity of sample sources, such as different occupations, different regions, etc. In terms of evaluation, a more scientific evaluation scale is adopted. At the same time, this study will increase the detection of the SIRT1 gene locus to further clarify the effect of the SIRT1 locus gene on type 2 diabetes comorbid depression.

## 5. Conclusion

To sum up, the patient’s gender, age, disease duration, marital status, income and drug expenditure, employment status, fasting blood glucose level, fasting insulin level, glycated hemoglobin, high-density lipoprotein level, and HCY were all important risk factors for patients with type 2 diabetes comorbid depression. SIRT1mRNA expression was strongly associated with the onset of type 2 diabetes comorbid depression. At the same time, there were differences in the genotype distribution frequencies of rs12415800, rs3758391, and rs932658 in the SIRT1 gene between patients with type 2 diabetes comorbid depression and patients with type 2 diabetes without depression. rs12415800 and rs3758391 were risk factors for type 2 diabetes comorbid depression, and rs932658 was a protective factor for type 2 diabetes comorbid depression. Furthermore, the findings suggest that the SIRT1 gene could serve as a potential diagnostic or therapeutic target. The identification of specific SNPs associated with type 2 diabetes comorbid depression paves the way for personalized medicine approaches, where SIRT1-targeted therapies might be developed to manage or mitigate the effects of this comorbidity. Future studies should explore practical applications of these findings in clinical settings to establish SIRT1 as a reliable tool for diagnosis and treatment.

## Author contributions

**Conceptualization:** Yingxia He, Qinqin Wu, Hong Zhu.

**Data curation:** Yingxia He, Qinqin Wu, Ziwei Yin, Hong Zhu.

**Formal analysis:** Yingxia He, Qinqin Wu, Hong Zhu.

**Investigation:** Ziwei Yin, Yi Zeng.

**Methodology:** Yi Zeng.

**Supervision:** Ningyu Xia.

**Writing – original draft:** Yingxia He, Hong Zhu.

**Writing – review & editing:** Yingxia He, Hong Zhu.
